# What is the Addictive Potential from Vaping?

**DOI:** 10.1177/14550725261437029

**Published:** 2026-04-03

**Authors:** Karl Erik Lund, Tord Finne Vedøy

**Affiliations:** 1 Department of Alcohol, Tobacco and Drugs, 25563 Norwegian Institute of Public Health, Oslo, Norway

**Keywords:** addiction, dependence, E-cigarettes, nicotine, smoking, snus, tobacco, vaping

## Abstract

**Aim:**

The present study discusses the addictive potential from vaping heuristically in view of existing research literature. We also paying attention to aayouth perspective in a Norwegian context.

**Methods:**

The study compiled paradigmatic explanations of addiction and then conducted a narrative review of the sparse literature on vape addiction. We draw som conclusions based on the results from a number of review-based viewpoints.

**Findings:**

Addiction to e-cigarettes or vapes is typically characterized by daily use, the first inhalation of the day occurring shortly after waking up, use of nicotine-containing e-liquid, continued use despite an expressed desire to abstain, acknowledgment that persistent use may lead to health harm and the experience of withdrawal symptoms following abrupt cessation. Higher frequency of use, longer duration of use, elevated nicotine concentration in the e-liquid, prior cigarette smoking and the use of pod-based e-cigarettes containing nicotine salts appear to be associated with a greater degree of dependence. Nicotine addiction among vapers may originate from previous smoking, although individuals without any smoking history also exhibit withdrawal symptoms. Overall, the level of addiction among vapers appears to be lower than that observed among smokers.

**Conclusions:**

Among Norwegian youth, e-cigarette use is generally experimental, transient and infrequent, and the use of nicotine-free products is common. It is therefore likely that a small segment of young vapers meets established criteria for addiction. Nevertheless, irregular use in the early stages may evolve into more dependency-driven patterns of use over time.

## Introduction

In a context where cigarette smoking among adolescents has declined substantially in most European countries, evidence suggests that the use of e-cigarettes – commonly referred to as vaping – is on the rise. In the Nordic countries, this increase occurs alongside a persistently high and stable prevalence of snus use (Nordic Welfare Centre, 2025). The observed increase in vaping, at least in Norway, can primarily be categorized as irregular and experimental use, which is often short-lived, and most common among young people who currently use or have previously used a tobacco product (Bakken, 2024; Tokle & Bakken, 2023, Tokle et al., 2022; Vedøy & Lund, 2025b).

Prospective studies indicate that the majority of irregular vapers remain low-intensity users (Roberts et al., 2024). However, for some, vaping may develop into more regular use and vaping has also increased among adolescents with no prior history of smoking. Health authorities in many countries have expressed concerns that e-cigarettes could perpetuate nicotine dependence across new generations and have therefore introduced strict regulatory measures, including flavor restrictions and bans on online sales. In Norway and Finland, the government's stated vision is a nicotine-free society (Linnansaari et al., 2022; Meld.St 15 (2022–2023), 2023).

While the potential harms to health associated with e-cigarette use have been examined in a number of systematic reviews (McNeill et al., 2022; National Academies of Sciences, Engineering and Medicine (NASEM), 2018; Royal College of Physicians, 2024; Valen et al., 2022), fewer studies have explored the addictive potential of e-cigarettes (Bold et al., 2018). To address this gap, the Library for Health Administration at the Norwegian Institute of Public Health (FHI) conducted a literature search for scientific papers published in English up until November 2023 applying keywords for outcome, intervention and populations respectively. Outcome terms were “addiction”, “nicotine dependence”, “nicotine reinforcement”, “abstinence”, “tobacco use disorder” and “withdrawal”. Intervention terms were “e-cigarettes”, “vaping”, “electronic nicotine delivery device (ENDS)”, “disposables”, “tanks”, “cig-a-likes” and “mods.” Population terms were “youth”, “teens”, “teenagers”, “adolescents”, “students” and “minors”. The search identified 319 articles. The procedure for the search strategy is shown in the Supplementary material (File S1).

Study titles and abstracts were screened by first investigator (KEL), with approximately 20% double-screened by the co-investigator (TFV). In case of disagreement, the studies were discussed based on a full-text screening followed by third party opinion.

The portfolio of studies was heterogeneous in terms of design, methodology, thematic focus and study populations. It therefore made little sense to apply rigorous exclusion and inclusion criteria, compare and rate the quality of studies, or to tabulate the studies into pre-exiting categories as required in a systematic review. Our assessment of study relevance was based on professional judgment established through decades in nicotine research and results are presented more like a narrative literature review (Ghosh & Choudhury, 2025; Gregory & Denniss, 2018). A narrative review is a flexible broad form of literature review that is used primarily when one wants to understand a research field, place research in context, or build theoretical arguments. The researcher pays attention to argumentation and interpretation, and is not steered by a rigorous method of source selection.

Approximately 200 studies were excluded because they did not focus on nicotine as an addictive agent. More specifically, the excluded studies typically focused on tobacco-related harm without any links to addiction mechanisms, on prevalence or descriptive epidemiology without any addiction-related analysis, on non-nicotine aspects of vaping (rituals or social meanings) without linking them to addiction, on results from highly technical molecular or animal studies without any clear transfer value to humans, on results from clinical cessation trials reporting quit rates only, on results from program evaluations lacking discussion of addiction mechanisms or because were opinion pieces or non-scholarly advocacy material.

The categorization of the remaining 120 studies followed a procedure where we let patterns and concepts emerge inductively during our reading (analog with the principles of grounded theory). The categories were studies comparing addiction between vaping and smoking, studies addressing nicotine concentration and addiction, studies addressing vaping device type and addiction and studies on self-declared addiction. Several of the studies could be included in multiple categories. A simple follow-up search was performed in October 2024 in PubMed and resulted in a small number of additional studies.

Other studies would perhaps have made a different selection/exclusion of studies and grouped the literature otherwise. Narrative literature reviews rely on interpretation, which can lead to researcher bias in the form of incomplete/uneven coverage, lack of transparency, lack of replicability, unclear inclusion/exclusion criteria, failure to assess study quality, risk of confirmation bias, etc. These biases, which are also present when conducting systematic reviews, might become a problem if they are not acknowledged. Despite limitations, our non-systematic narrative review is designed to achieved conceptual clarifications in the field and serve as an early-stage exploration that can shed light on various aspects of the addictive potential of e-cigarettes.

### Research Question

In the following, we first clarify the defining characteristics of substance addiction. We then review studies comparing addiction associated with vaping and smoking and discuss possible reasons for differences in the addictive potential of these products. Subsequently, we examine product-specific factors linked to varying levels of dependence, including nicotine concentration, e-cigarette device type and flavor profile. Finally, we attempt to reflect on a subgroup of adolescents in Norway who may potentielly be classified as having e-cigarette addiction according to the criteria that figure in the literature.

## Definitions of Addiction

Addiction is here used as a broad conceptual umbrella term for a complex phenomenon; definitions vary, and the concept is explained across different levels of abstraction and academic traditions. Several review articles (Cox, 2024; Field, 2024; Piper et al., 2006; Rise, 2014; West et al., 2024) and books (Bramness, 2018; Kraft, 2016) discuss the many conceptualizations of the term. To assess the addictive potential of e-cigarette use, it is necessary to land at some sort of understanding of how substance addiction is commonly defined.

### Neurobiological Perspectives

Neurobiological explanations of addiction are grounded in the notion that regular intake of a substance can result in physiological changes in the brain, leading to alterations in normal motivational processes through disruptions in neurotransmission or neuroadaptation – that is, the brain's adjustment to the effects of the substance. From this perspective, often referred to as the disease model, rational decision-making is overridden by automated reflexes, rendering substance use compulsive (Leshner, 1997; Volkow & Li, 2005). This approach has been criticized, among other reasons, for reducing the addicted individual to a passive observer of their own behavior.

### Motivational-Conflict Perspectives

An alternative explanatory tradition emphasizes that the addicted individual is caught in a motivational conflict between using and abstaining from using a substance. Here, the individual is capable of evaluating the pros and cons of their behavior, understanding and recognizing the consequences of their choices, yet remains unable to act in accordance with knowledge, insight and intention. Substance use continues despite a higher-order desire to quit – what the literature refers to as akrasia (weakness of will). Central to this explanatory framework are internal mental processes such as diminished willpower, present-oriented decision-making and reduced self-control (Elster & Skog, 1999; Gjelsvik, 1999; Heather, 2016).

### Behavioral and Descriptive Perspectives

Other researchers argue that addiction is best understood as a directly observable phenomenon, measurable by the frequency of substance intake, without recourse to underlying neurobiological mechanisms or the individual's ambivalent motivations (Rehm et al., 2013).

Some regard addiction as a normal feature of human behavior, insofar as we constantly succumb to temptation (Wakefield, 2020). Others emphasize a distinction between addiction and dependence or habit formation (O’Brien et al., 2006), whereas some consider such a distinction meaningless (Szalavitz et al., 2021). For certain researchers, addiction is viewed as a binary condition (one is either addicted or not), while others understand addiction as gradual or context-dependent (Lamontagne & Olmstead, 2019).

### Conceptual and Diagnostic Perspectives

Most lexical definitions highlight that substance addiction is typically associated with predictable harm to health (addictovocab.org, 2024), although some argue that adverse consequences need not be a defining criterion (Alexander & Schweighofer, 1988). Some conceptualizations describe addiction as involving a loss of free will, in the sense that decision-making is influenced by biologically driven processes that render substance use compulsive (Baumeister, 2017). Others maintain that, although the craving and temptation may be beyond voluntary control, the act of using or abstaining remains a matter of personal agency (Field, 2024; Skog, 2003).

Diagnostic systems such as the Diagnostic and Statistical Manual of Mental Disorders (DSM)-5 (American Psychiatric Association (APA), 2024; World Health Organization, 2024) are used in clinical practice to identify clinically significant substance use disorders. These frameworks assess symptoms such as tolerance, withdrawal, loss of self-control, social impairment, and health-related consequences. However, these diagnostic tools were primarily developed to assess the use of more intoxicating substances than nicotine (West et al., 2024).

Similaar to caffeine, which is also a psychoactive stimulant, nicotine has minimal impairing effects on perception and states of consciousness. Unlike alcohol and many narcotic substances, caffeine and nicotine do not impair reaction time, attention, memory, learning, judgment, speech, coordination or orientation. Consequently, within behavioral science, caffeine and nicotine are generally not classified as intoxicating drugs.

## Criteria of Addiction Applied to e-cigarettes

The terms “addiction” and “dependence” are often used without further distinction in the literature. Some consider that addiction should be reserved for behavior that results in foreseeable serious harm, and that this distinguishes addiction from dependence. When we refer to research in the present study, we have chosen to stick to the authors’ own terminology, knowing that this may obscure the distinction between the terms.

Drawing on conceptualizations of addiction/dependence proposed by some leading behavioral scientists (Elster & Skog, 1999; Gjelsvik, 1999; Rise, 2014; Skog, 2003) and applying them to e-cigarette use, the following characteristics may hypothetically be considered central to vape-addiction:

- Use of e-cigarettes occurs daily
- The first inhalation of the day occurs shortly after waking
- The vapor or e-liquid contains nicotine
- Use continues despite a strong desire to abstain
- The user acknowledges that persistent use may result in harm to health
- Abrupt cessation triggers withdrawal symptoms

These criteria may help identify the proportion of individuals within a given population – such as adolescents or young adults – who can be regarded as addicted to e-cigarettes. However, the signs of substance addiction typically develop gradually. The motives for experimenting with e-cigarettes differ from those that characterize continued use (Folivi et al., 2025) and users themselves often find it difficult to predict this development. Among adolescents, indications of e-cigarette addiction tend to emerge within 2–5 years after initiation (Adjei et al., 2024; Pienkowski et al., 2024), although some studies suggest that signs of addiction develop more rapidly (Graham-DeMello et al., 2024). Others contend that the addictive potential must be moderate, given that the majority are irregular vapers and remain low-intensity users (Roberts et al., 2024).

The reservoir of adolescents who may later in life develop addiction to e-cigarettes is likely far larger than the proportion who meet formal addiction (or dependency) criteria at an early stage. Moreover, the trajectory toward addictive-like use of e-cigarettes varies according to personality traits, mental health status (Manning et al., 2020; Masaki et al., 2022) and age of initiation (Maraqa et al., 2024).

## Studies Comparing Addiction to Vaping and to Smoking

For cigarette smoking, a range of self-report instruments have been developed to measure symptoms of dependence or addiction, such as the Wisconsin Inventory of Smoking Dependence Motives (WISDM-68), the Tobacco Dependence Index (TDI) and the Fagerström Test Score (FTS). The most widely used indicators of addiction in these measures are the “time to first cigarette after waking” (Heatherton et al., 1989; Kozlowski et al., 1981) and “self-reported craving intensity” (Dowd et al., 2019). Several qualitative (Afolalu et al., 2024; Simpson et al., 2021) and quantitative studies (Camenga et al., 2021; Piper et al., 2022; Rest et al., 2021; Soule et al., 2020) suggest that addiction to e-cigarettes should be assessed using modified versions of these instruments that are more appropriately tailored to vaping behavior.

In recent years, several such indices have been developed, including the Penn State Electronic Cigarette Dependence Index (PS-ECDI), the E-Cigarette Addiction Severity Index (EASI), the E-Cigarette Dependence Scale (EDS), the Roswell ENDS Nicotine Dependence Scale and the Adolescent and Young Adult Nicotine E-Cigarette Withdrawal Scale (AYA NEWS) (Foulds et al., 2015; Milstred et al., 2023; Morean et al., 2019a; Pienkowski et al., 2022; Piper et al., 2020; Sheffer et al., 2023; Vogel et al., 2020b).

However, there is no consensus on which scale best measures addiction/dependence, neither for smoking nor for vaping. This lack of standardization makes direct comparisons of addiction between the two forms of nicotine consumption challenging. Moreover, most adult users of e-cigarettes are former smokers (McNeill et al., 2022), and many vapers have thus established nicotine addiction through prior smoking (NASEM, 2018). Among young adults as well, vaping is most prevalent among individuals who currently use or have previously used tobacco products (Vedøy & Lund, 2025b).

Earlier reviews have noted that e-cigarette use – even among individuals with no prior history of smoking – can produce symptoms of nicotine dependence (Committee on Toxicity of Chemicals in Food, 2020; McNeill et al., 2018; NASEM, 2018). Based on the evidence available at the time, these reports concluded that both the risk of developing dependence and the severity of nicotine dependence appeared to be lower for vaping than for smoking. The NASEM report stated: “There is substantial evidence that e-cigarette use results in symptoms of dependence. There is moderate evidence that the risk and severity of dependence are lower for e-cigarettes than for combustible tobacco cigarettes” (NASEM, 2018; p. 7).

More recent studies published after these reviews confirm that vapers with no prior smoking experience can develop symptoms of nicotine withdrawal (Felicione et al., 2022; Parks et al., 2024), but overall addiction levels remain consistently lower among vapers than smokers when measured using the same indices (Boyd et al., 2022; Carpenter et al., 2023; Kaplan et al., 2020; Liu et al., 2017; Rhoades et al., 2019; Shiffman & Sembower, 2020; Strong et al., 2017, 2022, 2023; Vogel et al., 2020a). These findings are supported by studies employing alternative approaches (Etter & Eissenberg, 2015; Fagerström, 2018; Foulds et al., 2015; Hammond et al., 2021; Hughes & Callas, 2019; Jackson et al., 2021; Lohner et al., 2023; McNeill et al., 2022; Rycroft et al., 2021), although not all research concurs (Adjei et al., 2024; Jankowski et al., 2019; Rudasingwa et al., 2021). A recent review even found that the addictive potential of vaping and smoking was roughly equivalent (Kundu et al., 2025), although this conclusion was based on studies that did not adequately adjust for prior smoking experience among vapers.

Individuals who have replaced cigarettes with e-cigarettes generally report a lower degree of dependence on the latter compared to the former (Browne & Todd, 2018; Farsalinos et al., 2013, 2014; Hughes et al., 2020; Shiffman & Goldenson, 2023; Smith et al., 2020; Yingst et al., 2022). Dual users – those who alternate between e-cigarettes and traditional cigarettes – also tend to rate their dependence on e-cigarettes as lower (Perry et al., 2023). In general, the literature indicates that higher use intensity and concurrent tobacco use are associated with a greater number of dependence symptoms and higher dependence scores (Gomez et al., 2020; Kechter et al., 2021; Morean et al., 2019b; Strong et al., 2020).

Several studies have confirmed the rather self-evident relationship that both frequency of use (Singer et al., 2024; Xie et al., 2024) and duration of use are associated with greater addiction scores (Buu et al., 2021; NASEM, 2018; Vogel et al., 2020b). However, some studies have found that long-term e-cigarette users report a *decline* in perceived addiction over time (Etter, 2023), while others suggest that the state of dependence remains *stable* (Du et al., 2019). To summarize, although multiple instruments exist to assess nicotine dependence and newer scales have been adapted for vaping, the absence of a standardized measure, combined with the influence of prior smoking, complicates comparisons. Yet, the overall evidence indicates that e‑cigarette users might be exhibiting dependence symptoms at lower levels than cigarette smokers.

## Why do e-cigarettes Have a Lower Addictive Potential than Cigarettes?

Explanations for why e-cigarettes appear to have a lower addictive potential than combustible cigarettes may lie in differences in the amount of nicotine delivered and/or the presence of other substances that influence how rapidly psychoactive effects occur in the brain following intake.

Pharmacokinetic studies of vaping – that is, studies examining how nicotine is absorbed, distributed, and eliminated from the body – show that e-cigarettes generally produce both lower peak nicotine concentrations and lower overall nicotine exposure compared to cigarette smoking (Committee on Toxicity of Chemicals in Food, 2020; McNeill et al., 2022; NASEM, 2018). The time required to reach peak nicotine concentration is typically longer for e-cigarettes than for traditional cigarettes. However, experienced vapers may develop inhalation techniques that enable them to achieve nicotine concentrations comparable to those of smokers.

Another possible explanation is that tobacco smoke contains specific compounds that, either independently or in interaction with nicotine during combustion, enhance the urge for repeated use (Dybing & Sanner, 2002; Fagerström, 2018; Hogg, 2015; NASEM, 2018; Smith et al., 2016; Taylor et al., 2022; van der Toorn et al., 2019). Animal studies have shown that acetaldehyde – a chemical formed during the combustion of sugars added as sweeteners in tobacco – enhances the reinforcing properties of nicotine (Belluzzi et al., 2005; Hoffman & Evans, 2013). In the brain, nicotine binds to acetylcholine receptors, triggering the release of dopamine, serotonin, and adrenaline. Elevated dopamine levels in particular produce heightened sensations of pleasure and well-being.

## Nicotine Concentration and Addiction

As early as the first scientific reviews on e-cigarettes (McNeill et al., 2015), it was established that nicotine intake from e-cigarettes is highly variable and product specific. Based on the limited number of studies available at the time, these reports suggested that exposure to nicotine – and consequently the potential for addiction – increases with the use of e-liquids containing higher nicotine concentrations. More recent research has confirmed that the nicotine concentration in e-liquid can indeed influence the amount of nicotine absorbed into the bloodstream (Douglas et al., 2023). However, the relationship between the nicotine strength of the liquid and the actual nicotine uptake into the blood is complex.

One study demonstrated that biomarkers of nicotine exposure are only weakly correlated with the nominal nicotine strength of the e-liquid (Giberson et al., 2023) and that nicotine concentration did not vary in accordance with self-reported levels of dependence (Fearon et al., 2023). This lack of association may be explained by the fact that vapers, similar to smokers, self-regulate their nicotine intake through frequency and intensity of use – a phenomenon referred to as titration (Dawkins et al., 2016). Vapers who reduce the nicotine strength of their e-liquid over time, which is a common pattern of use (Soar et al., 2019), may compensate by increasing puff frequency and inhalation depth to maintain their desired nicotine intake. Experienced users often develop an efficient inhalation technique that can result in higher nicotine exposure than that of novice users operating the same device type (McNeill et al., 2022). Some researchers have therefore argued that imposing an upper cap on nicotine concentration in e-liquids may have unintended consequences in that vapers with particularly high nicotine needs will compensate through more intensive use, thereby increasing exposure to potentially harmful non-nicotine constituents of the vapor (Dawkins et al., 2016; McNeill et al., 2022).

The European Union's Tobacco Products Directive establishes an upper limit of 20 mg of nicotine per milliliter e-liquid (mg/ml) and a maximum container volume of 2 ml, corresponding to a total of 40 mg of nicotine. The majority of Norwegian vapers use e-liquids with nicotine concentrations well below 20 mg/ml. Between 2019 and 2022, 82% of users reported using e-liquids below this threshold (Vedøy & Lund, 2023).

The presence of nicotine is likely a necessary, but not sufficient, condition for the development of addiction. Researchers have also emphasized the importance of so-called “non-nicotine factors” in sustaining and reinforcing habitual use. These include behavioral and contextual rituals, tactile interactions with the device, and sensory cues such as taste and aroma (Andreas et al., 2024; Fagerström & Eissenberg, 2012; NASEM, 2018; Ou et al., 2024; Rose, 2006). To summarize, while nicotine concentration in e‑liquids plays a role in potential dependence processes, actual nicotine exposure seems to be shaped by complex patterns of self‑regulation, device use, and non‑nicotine behavioral cues, indicating that addiction to vaping cannot be understood solely in terms of nicotine strength.

## e-cigarette Type and Addiction

Earlier reports suggested that tank or modular devices deliver higher nicotine exposure and therefore possess greater addiction potential than cartridge-based or disposable models (McNeill et al., 2018; NASEM, 2018). More recent studies confirm that characteristics of the vaporizing unit influence nicotine uptake, primarily through the voltage output of the battery. An e-liquid with low nicotine concentration may deliver higher inhaled doses of nicotine if the battery voltage is increased, and vice versa (Do et al., 2022; Hoetger et al., 2022; Soule et al., 2023).

Compensatory behavior (titration) and technical features of the vaporizing unit mean that nicotine concentration in the e-liquid is not a valid indicator of actual nicotine absorption into the bloodstream. Consequently, attempts to establish equivalent measures – such as converting nicotine concentrations into cigarette-equivalent doses – are methodologically challenging and may produce misleading results.

In 2015, so-called “pod-type” e-cigarettes (e.g., Juul) were introduced in the USA with nicotine strengths of around 50 mg/ml. Unlike the European Union, the USA does not impose a regulatory cap on nicotine concentration. Pod systems are based on nicotine salts (protonated nicotine), in contrast to traditional e-cigarettes, which typically use freebase nicotine. Nicotine salts are produced by combining nicotine with an organic acid (Harvanko et al., 2019). Aerosol from nicotine salt-based e-cigarettes is perceived as smoother and less irritating to inhale (Cho et al., 2024; Harvanko et al., 2019; Leventhal et al., 2021), allowing users to tolerate higher nicotine concentrations with reduced throat discomfort (Leventhal et al., 2021). One study also found that the use of nicotine salt–based e-liquids was associated with inhaling larger aerosol volumes per puff (Talih et al., 2023).

Among adolescents using high-nicotine concentration pod systems, several studies have reported higher levels of dependence compared to users of other e-cigarette types (Boykan et al., 2019; Mantey et al., 2021; Sargent et al., 2022; Tackett et al., 2021). Among youth e-cigarette users in the USA, Canada and England, the proportion reporting symptoms of addiction increased between 2014 and 2021 (Gomes et al., 2024). Some scholars have speculated that this rise may be attributable to a market shift toward pod-based e-cigarettes (Glantz et al., 2022; Goniewicz et al., 2019; Hammond et al., 2023).

Pod use among adolescents is also prevalent in England. Approximately half of English 16–19-year-olds who had vaped within the past 30 days in 2021 reported using pod systems (McNeill et al., 2022). More recently, disposable pod-based e-cigarettes have gained popularity (Jackson et al., 2024). In England, as in the European Union, the nicotine concentration limit of 20 mg/ml remains in effect. Due to the relatively low voltage output of pod devices, nicotine absorption from British pods is lower than that of US products, where no such restriction currently exists. These contextual differences may explain why some studies have found minimal variation in dependence symptoms across different device types (Adjei et al., 2024; Douglas et al., 2023; Lin et al., 2022). One study, however, reported that users of tank-based, higher-powered devices (“mods”) exhibited greater dependence scores than users of slimmer, pod-based devices (Do et al., 2023).

At present, few studies have specifically examined whether particular flavorings are associated with the development of addiction. One study from 2022 found no such association (Sargent et al., 2022), whereas others have argued that flavor itself may exert an addictive or reinforcing influence (Gades et al., 2022).

In both the USA and Canada, vaporizing devices are also commonly used for cannabis consumption. In one Canadian study among young vapers, 55% reported having used e-cigarettes for cannabis liquids, while 32% reported exclusive use for this purpose (Dugas et al., 2020). Use of cannabis-containing e-liquids has been associated with higher nicotine dependence scores (Dugas et al., 2022).

Adolescents whose first nicotine product was an e-cigarette, report lower levels of dependence compared to those whose initiation into nicotine use occurred through snus use (Simon et al., 2023). All in all, the evidence reviewed for this study thus indicates that nicotine delivery and dependence risk vary considerably across device types, with technical features, regulatory contexts, and user behaviors shaping actual exposure far more than nominal nicotine strength alone.

## Self-Declared Addiction

Another approach to measuring addiction is to ask users to self-assess their perceived degree of dependence on e-cigarettes. In a British survey conducted in 2021, adolescents who had smoked or vaped within the past 30 days were asked whether they considered themselves addicted (McNeill et al., 2022). Among vapers, about half (53%) reported being addicted (“yes, somewhat addicted” or “yes, very addicted”), while slightly fewer (43%) reported not being addicted at all. The remaining 4% were uncertain. The proportion identifying as addicted was more than three times higher among vapers who were current (64%) or former (74%) cigarette smokers than among vapers with no prior smoking history (20%). Among smokers, a large majority (83%) reported being “somewhat” or “very” addicted to cigarettes, a minority (15%) said “not at all” and only 3% were uncertain (McNeill et al., 2022).

In a US study of young regular vapers, approximately half reported not feeling addicted at all, 41% considered themselves “somewhat addicted” and 13% viewed themselves as “addicted” (Camara-Medeiros et al., 2021).

Another commonly used indicator of dependence is the frequency of cravings to vape or smoke. In the British survey by McNeill et al. (2022), 21% of young vapers reported experiencing urges to vape several times a day, an additional 24% experienced daily cravings, and 21% said that cravings occurred weekly. The remaining 31% reported rarely or never experiencing any urge to vape. Among young smokers, cravings occurred more frequently: 35% several times a day, 32% daily, 19% weekly and 13% rarely or never (McNeill et al., 2022).

A related indicator of addiction is the self-reported intensity of craving. In the above mentioned British study, regular vapers aged 11–18 years described their urge to use e-cigarettes as extremely strong (1%), very strong (10%) or strong (13%). A greater proportion reported moderate (24%), slight (12%) or negligible (42%) cravings. In comparison, smokers reported stronger urges: extremely strong (4%), very strong (11%), strong (16%), moderate (26%), slight (18%) and negligible (24%) (McNeill et al., 2022). Another key finding was that addiction to e-cigarettes was greater among vapers who currently or previously smoked cigarettes, compared to those without any smoking history. To summarize, self‑reported assessments show that perceived addiction and craving intensity are lower among vapers than among smokers, especially for those without a prior smoking history. This should naturally also be viewed in the light of common validity questions surrounding self-estimation.

## Are We Seeing a New Generation of Nicotine-Addicted Youth?

Public policy documents on tobacco control have expressed a concern that the diversification of the nicotine market could create a product landscape that entices new generations into nicotine addiction (Meld. St. 15 (2022–2023), 2023; p. 45). Nicotine addiction is in itself undesirable, but the degree of harm can depend on the route of nicotine administration (McNeill et al., 2022). Between 1950 and 2000, almost all nicotine was consumed through the inhalation of smoke from the combustion of tobacco (pipes, roll-your-own and manufactured cigarettes) (Lund & Vedøy, 2023). This form of nicotine intake caused approximately two out of three long-term users to die from smoking-related diseases, primarily lung cancer, chronic obstructive pulmonary disease and cardiovascular conditions (Doll et al., 2004; Pirie et al., 2013). Governmental efforts to curb smoking-related harm began in the late 1960s (Lund & Vedøy, 2024). Over the subsequent decades, the fight against smoking became synonymous with the fight against nicotine. Consequently, the expression “a new generation of nicotine addicts” evokes associations to the extensive harms experienced by earlier generations of nicotine users – namely cigarette smokers.

According to the Royal College of Physicians, however, nicotine dependence through e-cigarettes “exposes users to a much narrower range of toxins than smoking, and the levels of toxins absorbed from vaping are generally low. It is therefore likely that vaping poses only a small fraction of the risk of smoking” (Royal College of Physicians, 2024; p. 67).

The forthcoming repeal of Norway's ban on nicotine-containing e-liquids, scheduled for implementation in 2025, has fueled concerns about a new generation of nicotine-dependent youth. Yet the nicotine content itself is not the main driver of e-cigarette use among adolescents. Unlike established smokers, youth without prior tobacco experience have no inherent physiological craving for nicotine. Instead, young people cite other motives for initiating e-cigarette use, such as flavor, curiosity, the appeal of transgressive behavior, identity expression, group affiliation and the performative aspect of exhaling large clouds of vapor (Kinouani et al., 2025; Tokle & Scheffels, 2024).

The recent rise in e-cigarette use among Norwegian youth, primarily experimental and irregular use, has occurred during a period when the sale of nicotine-containing e-liquids has been prohibited. Continued growth in youth vaping is plausible but would likely occur regardless of whether the nicotine ban is lifted or not. However, lifting the ban could lead to more frequent experimentation with nicotine-containing products, thereby increasing the potential risk of developing dependence.

Applying the addiction criteria discussed earlier, the use of nicotine-free e-cigarettes, as well as experimental, transient, or low-frequency use of nicotine-containing e-cigarettes, would fall outside the definition of addiction. This implies that only a small group of Norwegian youth could be characterized as potentially addicted.

According to standard criteria, addiction-related behavior would also be present if these individuals continued vaping despite a first-order intention to abstain - an instance of the aforementioned akrasia. There is currently no research identifying the size of the subgroup of young people who continue to vape against their own will.

If vaping persists despite an awareness of potential health risks, another criterion for addiction is fulfilled. However, young vapers generally perceive e-cigarettes as moderately harmful. In the European School Survey Project on Alcohol and Other Drugs (ESPAD), Norwegian adolescents (aged 15 and 16 years) were asked how harmful they thought e-cigarettes with nicotine was. In 2024, 23% of regular vapers (*n* = 344) answered that they believed regular vaping with nicotine was a “large risk”. In contrast, 60% of regular smokers (*n* = 145) believed smoking a pack of cigarettes a day constituted a “large risk” (Vedøy & Bye, 2025). Consequently, a higher proportion of smokers than vapers would meet this particular addiction criterion.

If cessation of vaping were to induce withdrawal symptoms, such as irritability, aggression, low mood, anxiety, restlessness or difficulty concentrating, this would indicate dependence. There are currently no Norwegian data on the prevalence of withdrawal symptoms following e-cigarette cessation. However, studies primarily from the USA show that vapers do indeed report withdrawal symptoms, although their frequency, character and intensity vary (Browne & Todd, 2018; Buu et al., 2021; Du et al., 2019; Etter, 2023; Farsalinos et al., 2013, 2014; Felicione et al., 2022; Gomez et al., 2020; Hughes et al., 2020; Kechter et al., 2021; Morean et al., 2019b; Perry et al., 2023; Shiffman & Goldenson, 2023; Smith et al., 2020; Strong et al., 2020; Vogel et al., 2020a).

Although current patterns of e-cigarette use among Norwegian youth suggest that a small proportion meet the criteria typically defining addiction, those who maintain sustained use may develop some kind of dependence over time. The potential loss of autonomy, financial costs and health detriments associated with nicotine addiction through e-cigarettes are nonetheless undesirable and youth vaping should be actively prevented.

## Conclusions

Based on the processual conclusions drawn in this conceptual narrative review, we suggest that in populations with a high prevalence of smoking, e-cigarettes could function as a harm-reducing, although not risk-free, alternative to combustible tobacco. Among Nordic youth, however, cigarette smoking has become increasingly rare, rendering e-cigarettes largely obsolete as a cessation aid for this group. Although the use of snus or nicotine pouches is widespread among young people in the Nordic countries, the risk differential between these oral products and vaping is considered to be relatively small, meaning that e-cigarettes do not represent a meaningful harm-reduction alternative for users of oral nicotine products.

For youth at elevated risk of smoking initiation, e-cigarettes may represent a less harmful alternative. Yet, this subgroup is likely small. For the majority of adolescents, as well as those without the traits typically associated with smoking initiation, e-cigarette use may introduce health risks and traits of dependence that they would otherwise have avoided. Current use patterns among youth suggest that a small segment exhibit behaviors consistent with addiction, although the reservoir of young users who may later develop signs of dependence remain significantly larger. Compared with tobacco smoking, research consistently indicates that the addictive potential of vaping is lower.

## Supplemental Material

sj-docx-1-nad-10.1177_14550725261437029 - Supplemental material for What is the Addictive Potential from Vaping? Supplemental material, sj-docx-1-nad-10.1177_14550725261437029 for What is the Addictive Potential from Vaping? by Karl Erik Lund and Tord Finne Vedøy in Nordic Studies on Alcohol and Drugs
