# Supplementary material for: What is the Addictive Potential from Vaping?
Source: Nordisk Alkohol Nark. 2026 Apr 3;43(3):325–45. doi: 10.1177/14550725261437029 (PMC13048976; doi:10.1177/14550725261437029)
Supplement: sj-docx-1-nad-10.1177_14550725261437029 - Supplemental material for What is the Addictive Potential from Vaping? [file sj-docx-1-nad-10.1177_14550725261437029.docx]

**Supplementary file 1**

**Literature search**

The electronic databases below were searched. The initial search was conducted on 6/11/2023 and was limited to English-language studies only. Boolean sample search string is outlined in Table 1 below. After duplicate check, 319 references were identified.

**Database**: Ovid MEDLINE(R) and Epub Ahead of Print, In-Process, In-Data-Review

& Other Non-Indexed Citations, Daily and Versions <1946 to November 03, 2023>.

Number of references identified: 169

| 1 | Electronic Nicotine Delivery Systems/ or (((electronic or e) adj cig*) or ecig* or "electronic nicotine delivery system?").tw,kf. | 11581 |
| --- | --- | --- |
| 2 | Vaping/ or ((nicotin* adj1 vapo*) or ((e or electronic or pen?) adj1 hookah?)).tw,kf. | 3821 |
| 3 | 1 or 2 | 11939 |
| 4 | "Tobacco Use Disorder"/ or (addict* or dependen* or ((tobacco or nicotin*) adj ("use disorder?" or abuse))).tw,kf. | 2008736 |
| 5 | Adolescent/ or Students/ or Minors/ | 2273452 |
| 6 | (adolescen* or preadolescen* or preteen? or tween? or tweenager? or juvenil* or under age* or underage* or teen? or teenager? or minor* or pubescen* or (young adj (people* or person*)) or youth* or student* or (((age or aged) adj ("10" or ten or "11" or eleven or "12" or twelve or "13" or thirteen or "14" or fourteen or "15" or fifthteen or fifteen or "16" or sixteen or "17" or seventeen or "18" or eighteen or "19" or nineteen)) or (("10" or ten or "11" or eleven or "12" or twelve or "13" or thirteen or "14" or fourteen or "15" or fifthteen or fifteen or "16" or sixteen or "17" or seventeen) adj ("year* old" or "yr* old")))).tw,kf. | 1494541 |
| 7 | 5 or 6 | 3175062 |
| 8 | (((prevalence or symptom?) adj6 dependen*) or ((addiction or abuse) adj liabilit*) or ((dependen* or abstinence) adj (symptom? or motive? or potential? or rating? or index* or score?)) or ((risk? or characteristic? or nature or level? or measur* or asses* or associat* or change?) adj3 dependen*) or ((less or very or more) adj (addictive or dependen*)) or (potential adj2 abuse)).tw,kf. | 127056 |
| 9 | 3 and 4 and 7 and 8 | 212 |
| 10 | limit 9 to yr="2019 -Current" | 169 |
| 11 | limit 10 to (danish or english or norwegian or swedish) | 169 |

**Database**: Embase <1974 to 2023 November 03>.

Number of references identified: 224

| 1 | electronic cigarette/ or (((electronic or e) adj cig*) or ecig* or "electronic nicotine delivery system?").tw,kf. | 14438 |
| --- | --- | --- |
| 2 | vaping/ or ((nicotin* adj1 vapo*) or ((e or electronic or pen?) adj1 hookah?)).tw,kf. | 6048 |
| 3 | 1 or 2 | 15703 |
| 4 | tobacco dependence/ or (addict* or dependen* or ((tobacco or nicotin*) adj ("use disorder?" or abuse))).tw,kf. | 2399844 |
| 5 | exp Adolescence/ or exp Adolescent/ or juvenile/ or student/ or middle school student/ or high school student/ or minor/ | 1963056 |
| 6 | (adolescen* or preadolescen* or preteen? or tween? or tweenager? or juvenil* or under age* or underage* or teen? or teenager? or minor* or pubescen* or (young adj (people* or person*)) or youth* or student* or (((age or aged) adj ("10" or ten or "11" or eleven or "12" or twelve or "13" or thirteen or "14" or fourteen or "15" or fifthteen or fifteen or "16" or sixteen or "17" or seventeen or "18" or eighteen or "19" or nineteen)) or (("10" or ten or "11" or eleven or "12" or twelve or "13" or thirteen or "14" or fourteen or "15" or fifthteen or fifteen or "16" or sixteen or "17" or seventeen) adj ("year* old" or "yr* old")))).tw,kf. | 1960133 |
| 7 | 4 or 5 | 4289320 |
| 8 | (((prevalence or symptom?) adj6 dependen*) or ((addiction or abuse) adj liabilit*) or ((dependen* or abstinence) adj (symptom? or motive? or potential? or rating? or index* or score?)) or ((risk? or characteristic? or nature or level? or measur* or asses* or associat* or change?) adj3 dependen*) or ((less or very or more) adj (addictive or dependen*)) or (potential adj2 abuse)).tw,kf. | 155251 |
| 9 | 3 and 4 and 7 and 8 | 480 |
| 10 | limit 9 to yr="2019 -Current" | 333 |
| 11 | limit 10 to (danish or english or norwegian or swedish) | 333 |
| 12 | limit 11 to embase | 224 |

**Database**: Web of Science Core Collection, Science Citation Index Expanded (SCI-EXPANDED)--1987-present, Social Sciences Citation Index (SSCI)--1987-present, Arts & Humanities Citation Index (AHCI)--1987-present, Emerging Sources Citation Index (ESCI)--2018-present

Number of references identified: 160

| 8 | #3 AND #4 AND #5 AND #6 and 2019 or 2020 or 2021 or 2022 or 2023 (Publication Years) | \| Exact search | 160 |
| --- | --- | --- | --- |
| 7 | #3 AND #4 AND #5 AND #6 | \| Exact search | 204 |
| 6 | TS=(((prevalence or symptom$) NEAR/5 dependen*) or ((addiction or abuse) NEAR/0 liabilit*) or ((dependen* or abstinence) NEAR/0 (symptom$ or motive$ or potential$ or rating$ or index* or score$)) or ((risk$ or characteristic$ or nature or level$ or measur* or asses* or associat* or change$) NEAR/2 dependen*) or ((less or very or more) NEAR/0 (addictive or dependen*)) or (potential NEAR/1 abuse)) | \| Exact search | 195,713 |
| 5 | TS=(adolescen* or preadolescen* or preteen$ or tween$ or tweenager$ or juvenil* or "under age*" or underage* or teen$ or teenager$ or minor* or pubescen* or (young NEAR/0 (people* or person*)) or youth* or student* or (((age or aged) NEAR/0 ("10" or ten or "11" or eleven or "12" or twelve or "13" or thirteen or "14" or fourteen or "15" or fifthteen or fifteen or "16" or sixteen or "17" or seventeen or "18" or eighteen or "19" or nineteen)) or (("10" or ten or "11" or eleven or "12" or twelve or "13" or thirteen or "14" or fourteen or "15" or fifthteen or fifteen or "16" or sixteen or "17" or seventeen or "18" or eighteen or "19" or nineteen) NEAR/0 ("year* old" or "yr* old")))) | \| Exact search | 2,321,493 |
| 4 | TS=(addict* or dependen* or ((tobacco or nicotin*) NEAR/0 ("use disorder$" or abuse))) | \| Exact search | 3,346,365 |
| 3 | #1 or #2 | \| Exact search | 12,491 |
| 2 | TS=((nicotin* NEAR/0 vapo*) or ((e or electronic or pen$) NEAR/0 hookah$)) | \| Exact search | 212 |
| 1 | TS=(((electronic or e) NEAR/0 cig*) or ecig* or "electronic nicotine delivery system$") | \| Exact search | 12,408 |
